# Doctor Fell

**Published:** 1910-01

**Authors:** 


### Doctor Fell.

I do not love you, Doctor Fell; the reason why, I'll briefly tell: The doctor of the olden days had kindly words and pleasant ways; and though his pills were on the bum, and sent folks off to Kingdom Come, and though he liked to swell the hosts of skeletons and sheeted ghosts, it never was his foolish plan to use a saw on every man. Unlike the modern maniacs, who carve their patients with an axe, he dealt out calomel or nux, and soaked us for a pair of bucks; and if he killed us—good old soul! he left us to be planted whole.

When I am sickly and unstrung, you ask me to unfurl my tongue; you feel my pulse and prod my back, and say my liver's out of whack, and then you shed your vest and coat, and push a lantern down my throat, and say: "Great Cæsar! What a heart! I'll have to take you all apart." And on your table I am laid, while you go out to hunt a spade, to dig around among my works and find the blamed old germ that lurks around the angles of my frame—the way you carve me is a shame.

When winter comes, with frost and snow, I have a chilblain on my toe; and when for liniment I beg, you want to amputate my leg; and when my throat gets sore and raw, you want to cure it with a saw; to cure my baldness, you, I ween, would run me through a guillotine. A leg of mine is now at rest among the doctors of the West; an Eastern doctor has in brine about eight inches of my spine; the jaw that once adorned my mouth, is kept in pickle in the South.

I do not love you, Doctor Fell; you carve too fluently and well; I fear you and your edged tools; I'll send to correspondence schools for absent treatment when I'm ill—or hit the good old-fashioned pill.—*Walt Mason in the Emporia Gazette.*

